# From pathophysiology to therapy: molecular mechanisms of stem cell and extracellular vesicle-mediated repair in diabetic peripheral neuropathy

**DOI:** 10.3389/fcell.2026.1854350

**Published:** 2026-06-16

**Authors:** Jiayi Zhou, Yang Yu, Liuchang Song, Peipei Qian, Mengni Zhao, Fei Ding, Xuegan Lian, Miaomei Yu

**Affiliations:** 1 Clinical Medical Research Center and Department of Neurology, The Third Affiliated Hospital of Soochow University, Changzhou, Jiangsu, China; 2 Jiangsu Key Laboratory of Tissue Engineering and Neuroregeneration, Key Laboratory of Neuroregeneration of Ministry of Education, Co-Innovation Center of Neuroregeneration, NMPA Key Laboratory for Research and Evaluation of Tissue Engineering Technology Products, Nantong University, Nantong, Jiangsu, China

**Keywords:** cell-free therapy, diabetic peripheral neuropathy, extracellular vesicles, molecular mechanisms, nerve regeneration, stem cell therapy

## Abstract

Diabetic peripheral neuropathy (DPN) is a prevalent diabetic complication with limited effective treatments. While stem cell-based therapies show promise, their efficacy is attributed to paracrine activity, particularly extracellular vesicles (EVs). This review examines the molecular pathology of DPN, focusing on metabolic dysregulation, neuroinflammation, and neurovascular dysfunction. The mechanisms by which stem cells and their EVs counteract these processes are elucidated, including activating antioxidant pathways (NRF2/HO-1), reprogramming immune signaling (M1 to M2 polarization), and promoting neurovascular regeneration (PI3K/Akt, MAPK). Innovations in EV engineering aimed at enhancing their molecular cargo and targeting specificity are critically assessed. Furthermore, the translational challenges for these EV-based therapies are addressed, focusing on the need for standardized production to ensure molecular consistency. It is concluded that EV-based cell-free therapy represents a novel regenerative strategy for DPN, operating through multi-targeted molecular mechanisms.

## 1 Introduction

Diabetic peripheral neuropathy, a debilitating chronic complication of diabetes, is characterized by distal symmetric polyneuropathy, leading to sensory loss, neuropathic pain, and a high risk of lower-limb amputations, thereby severely impacting patient quality of life (Savelieff et al., 2025). With its global prevalence rising, DPN affects a significant proportion of diabetic patients, underscoring an urgent unmet clinical need for therapies that can reverse, rather than merely manage, nerve damage (Savelieff et al., 2025; Yang et al., 2025). Current clinical management of DPN primarily revolves around three strategies: intensive glycemic control, symptomatic relief of neuropathic pain, and neurotrophic pharmacological interventions. However, each has substantial limitations. While intensive glycemic control reduces the risk of DPN in type 1 diabetes, its benefits are less pronounced in type 2 diabetes, and it cannot completely halt progressive neurodegeneration (Didangelos et al., 2021; Jang and Oh, 2023; Lee and Won, 2025). Pain management, involving anticonvulsants, antidepressants, and topical agents, provides only partial relief and is often accompanied by dose-limiting adverse effects. Neurotrophic agents such as alpha-lipoic acid and mecobalamin may modestly alleviate symptoms but have not been shown to reverse established nerve damage. Non-pharmacological interventions such as foot care, physical therapy, and acupuncture mainly lie in preventing ulcers and deformities, with no reversal effect on the neuropathy itself (Aziz et al., 2025; Baicus et al., 2024; van Netten et al., 2024; Zhou et al., 2023). These limitations underscore the urgent need for innovative therapies with neural regenerative capacity.

The pathogenesis of DPN is a multifactorial process driven by chronic hyperglycemia. It involves a complex interplay of metabolic disturbances, oxidative stress, chronic inflammation, and microvascular dysfunction, ultimately culminating in the degeneration of nerve fibers. At the molecular level, these pathological processes are orchestrated by a network of interconnected signaling pathways, including the activation of the polyol pathway, formation of advanced glycation end-products (AGEs) and their receptor for advanced glycation end-products (RAGE), protein kinase C (PKC) activation, mitochondrial dysfunction leading to reactive oxygen species (ROS) overproduction, and persistent activation of pro-inflammatory cascades like nuclear factor kappa-light-chain-enhancer of activated B cells (NF-κB) and mitogen-activated protein kinases (MAPKs) (Abdelkader et al., 2022; Aghamiri et al., 2022; Eftekharpour and Fernyhough, 2022; Eid et al., 2022; Fan B. et al., 2020; Pathak et al., 2022). Understanding this intricate molecular pathology is crucial for developing targeted and effective therapies.

In recent years, stem cell-based therapies have emerged as a promising frontier for DPN, demonstrating potential for tissue repair and immunomodulation (Alizadeh et al., 2024). However, a paradigm shift has occurred with the growing recognition that the therapeutic benefits of stem cells are largely mediated by their paracrine activity, particularly through the secretion of extracellular vesicles (EVs) (Feng et al., 2023; Wei et al., 2025). EVs are a heterogeneous group of lipid bilayer-enclosed particles broadly classified into exosomes (small EVs, 50–150 nm), microvesicles (50–1,000 nm), and apoptotic bodies (>1,000 nm), with exosomes being the most extensively studied subtype in DPN therapy (Lu et al., 2025; van Niel et al., 2018). These stem cell-derived EVs act as natural nanoscale delivery vehicles, shuttling a complex cargo of bioactive molecules to recipient cells, including proteins, lipids, and nucleic acids such as microRNAs (miRNAs) (Draguet et al., 2023; Herrmann et al., 2021; Serrano et al., 2025). By transferring these molecular signals, EVs can modulate key pathological pathways in neurons, Schwann cells (SCs), and immune cells, thereby exerting neuroprotective, anti-inflammatory, and pro-regenerative effects (Namini et al., 2023; Shi et al., 2025; Tajabadi et al., 2025; Zhang et al., 2025). This positions EVs as a highly promising cell-free therapeutic strategy, inheriting the reparative potential of their parent cells while offering advantages such as low immunogenicity and enhanced engineerability (Izquierdo-Altarejos et al., 2024; Namini et al., 2023).

This review aims to provide a comprehensive and mechanistic overview of how stem cells and their EVs restore neural function in DPN. We will systematically delineate the key molecular pathways driving DPN pathology and then explore, in depth, the specific molecular mechanisms through which stem cell-derived EVs exert their therapeutic effects. This includes their role in counteracting oxidative stress *via* pathways like nuclear factor erythroid 2-related factor 2/heme oxygenase 1 (NRF2/HO-1), reprogramming neuroinflammation by modulating toll-like receptor 4 (TLR4)/NF-κB and microglial wingless-type MMTV integration site family, member 5a/transient receptor potential vanilloid type 1 (WNT5a/TRPV1) signaling, and promoting coordinated neurovascular regeneration through the delivery of growth factors and miRNAs that activate survival and regenerative pathways like phosphoinositide 3-kinase/AKT serine/threonine kinase (PI3K/Akt) and MAPK (Chen et al., 2023; Chen et al., 2025; Fan et al., 2021; Shan et al., 2022; Wang L. et al., 2025). Furthermore, we will critically assess recent advances in EV engineering designed to enhance their molecular cargo and targeting capabilities, and evaluate the translational landscape and challenges for advancing these molecularly targeted, EV-based therapies toward clinical application in DPN.

## 2 Clinical diagnosis and pathophysiological basis of DPN

### 2.1 Clinical manifestations and diagnostic criteria

DPN, as a prototypical symmetric, length-dependent sensorimotor polyneuropathy, presents with complex and diverse clinical manifestations involving sensory, motor, and autonomic nerve dysfunction (Lee and Won, 2025). Symptoms typically begin in the distal extremities, exhibiting a characteristic pattern of sensory impairment: severe neuropathic pain (e.g., burning, electric-shock, or stabbing pain), often worsening at night and severely disrupting sleep, dominates the early stage. The mid-stage features paresthesia, such as hyperalgesia, thermal sensation abnormalities, and formication; whereas late-stage progresses to sensory loss, with diminished or absent perception of pain, temperature, and touch, significantly increasing the risk of painless foot ulcers, infection, and ultimately amputation. Motor dysfunction similarly manifests as distal, progressive muscle weakness and atrophy, particularly affecting ankle dorsiflexion, leading to a characteristic steppage gait and substantially elevating fall risk (Yang et al., 2022). Furthermore, autonomic involvement may affect multiple systems, presenting as cardiovascular regulation abnormalities (e.g., orthostatic hypotension, resting tachycardia), gastrointestinal symptoms (e.g., gastroparesis, bowel dysfunction), genitourinary dysfunction, and sweating abnormalities, further amplifying disease burden (Bell, 2023; Eleftheriadou et al., 2024; Jia et al., 2025).

Due to its complex and overlapping clinical presentation, DPN diagnosis necessitates a multimodal assessment (Dillon et al., 2024; Yang et al., 2022). Core diagnostic criteria include: (1) confirmed diabetes or evidence of impaired glucose metabolism; (2) typical clinical symptoms of DPN as described above; and (3) objective abnormalities in at least two neurophysiological or structural tests. Nerve conduction velocity (NCV) remain the key tool for assessing large fiber nerve function; however, their sensitivity is limited in small fiber neuropathy, which is predominantly characterized by pain and autonomic symptoms, necessitating more refined techniques such as skin biopsy for evaluating intraepidermal nerve fiber density (IENFD) (Devigili et al., 2025). Additionally, high-resolution ultrasound and magnetic resonance imaging (MRI) can visualize nerve structural alterations (Zhang and Zhang, 2023). In clinical practice, combining screening tools and scoring scales, such as the Michigan Neuropathy Screening Instrument (MNSI), enhances detection efficiency and the accuracy of severity grading. Emerging technologies, including artificial intelligence (AI)-based retinal image analysis, show promise for noninvasive DPN risk assessment and represent a potential future direction in diagnosis.

Early diagnosis and intervention are crucial for preventing serious complications like foot ulcers, infection, and amputation. Hence, routine neurological evaluation should commence at diagnosis for type 2 diabetes and within 5 years for type 1 diabetes (Bell and Jerkins, 2025). For refractory DPN, particularly in patients with long disease duration and poor response to conventional therapies (e.g., antioxidants, aldose reductase inhibitors), diagnosis emphasizes chronicity and treatment resistance, underscoring the urgent need for novel therapeutic strategies such as stem cell- and EV-based interventions. Integrating multifactorial risk models with multimodal diagnostic approaches to achieve early, precise DPN identification is pivotal for optimizing clinical management and improving patient outcomes (Pathak et al., 2022).

### 2.2 Pathophysiological mechanisms

#### 2.2.1 Metabolic disturbances and oxidative stress: a molecular cascade

Hyperglycemia serves as the primary metabolic insult initiating DPN, triggering a cascade of interconnected molecular pathways that culminate in cellular dysfunction and oxidative stress (Lin et al., 2023). At the molecular level, excess glucose flux activates the polyol pathway, where aldose reductase converts glucose to sorbitol, consuming reduced nicotinamide adenine dinucleotide phosphate (NADPH) and depleting the cellular antioxidant glutathione (GSH) (Pathak et al., 2022). Concurrently, hyperglycemia drives the non-enzymatic formation of AGEs, which bind to their receptor RAGE, activating the transcription factor NF-κB and promoting the expression of pro-inflammatory cytokines and ROS-generating enzymes (Abdelkader et al., 2022). Hyperactivity of the PKC-β isoform impairs vascular function by reducing nitric oxide (NO) bioavailability. A central hub of this metabolic dysregulation is the mitochondrion; hyperglycemia-induced overproduction of electron donors disrupts the electron transport chain, leading to a massive increase in ROS and reactive nitrogen species (RNS). This exceeds the capacity of endogenous antioxidant systems like superoxide dismutase (SOD), causing oxidative damage to lipids (e.g., malondialdehyde, MDA), proteins, and DNA (Eftekharpour and Fernyhough, 2022; Eid et al., 2022). The resulting oxidative stress is intimately linked with endoplasmic reticulum (ER) stress, where the accumulation of unfolded proteins activates the inositol-requiring enzyme 1α-C/EBP homologous protein (IRE1α-CHOP) pro-apoptotic axis, leading to Caspase-3/12 activation and apoptosis of SCs and dorsal root ganglion (DRG) neurons (Zhu et al., 2021). Understanding these oxidative and ER stress pathways is clinically relevant because they represent early, druggable targets for interrupting DPN progression before irreversible nerve damage occurs. Antioxidant strategies that restore redox balance or alleviate ER stress may therefore offer disease-modifying potential beyond symptomatic relief.

#### 2.2.2 Chronic neuroinflammation and immune dysregulation: signaling pathways in the nervous system

DPN progression is marked by a state of chronic neuroinflammation driven by a persistent cycle of molecular signals (Cheng et al., 2024; Fan et al., 2021). Metabolic stress acts as a danger signal, activating immune cells like macrophages, which release pro-inflammatory cytokines tumor necrosis factor-α (TNF-α), interleukin-1β (IL-1β), and interleukin-6 (IL-6) (Hinder et al., 2018). These cytokines, in turn, activate key pro-inflammatory signaling pathways within neurons and glia, including the NF-κB, MAPKs, and Toll-like receptors (TLRs) pathways, thereby amplifying the inflammatory cascade and exacerbating neural injury (Aghamiri et al., 2022; Fan B. et al., 2020). In the spinal cord dorsal horn, microglia and astrocytes are activated under diabetic conditions. This activation is coupled with metabolic reprogramming; for instance, hyperglycemia suppresses Sirtuin 3 (Sirt3) in microglia *via* the Akt/forkhead box O1 (FOXO1) axis, shifting their metabolism toward aerobic glycolysis and enhancing pro-inflammatory mediator production (Li et al., 2024). The release of TNF-α and IL-1β directly drives neuronal hyperexcitability and central sensitization, underlying neuropathic pain. Aberrant activation of the WNT5a/TRPV1 signaling axis in microglia further promotes their polarization toward the pro-inflammatory M1 phenotype, aggravating local inflammation (Chen et al., 2025). Elucidating these inflammatory signaling cascades matters because modulating microglial polarization, for instance, shifting from the pro-inflammatory M1 to the anti-inflammatory M2 phenotype, offers a promising therapeutic strategy to alleviate neuropathic pain and halt neural degeneration. Targeting WNT5a/TRPV1 or NF-κB pathways could therefore yield novel disease-modifying interventions for painful DPN.

#### 2.2.3 Structural disintegration and dysfunction of the neurovascular unit (NVU): from cellular crosstalk to molecular signals

The NVU, comprising neurons, glia (especially SCs), and vascular cells, is essential for nerve homeostasis. In diabetes, its integrity is disrupted through a cascade of molecular aberrations. Axonal degeneration, a hallmark of DPN, is mediated by a molecular pathway involving sterile alpha and TIR motif containing 1 (SARM1), which triggers nicotinamide adenine dinucleotide (NAD^+^) depletion, pathological calcium influx, and mitochondrial dysfunction (Chen et al., 2021). SCs are central to NVU function, and diabetic conditions impair SC physiology, reducing proliferative and repair capacity, increasing apoptosis, and blocking the pro-regenerative phenotype. These impairments result from metabolic stress (polyol pathway flux and AGE formation), oxidative damage (ROS accumulation exceeding antioxidant capacity), ischemia (microvascular dysfunction leading to nerve hypoxia), and inflammatory cytokines (TNF-α, IL-1β, IL-6 released from activated macrophages and glial cells) (Abdelkader et al., 2022; Eftekharpour and Fernyhough, 2022; Eid et al., 2022; Fan B. et al., 2020; Goldney et al., 2023; Group et al., 2022; Hinder et al., 2018; Pathak et al., 2022; Wang et al., 2025b). Under high-glucose conditions, SCs secrete exosomes enriched with specific miRNAs (e.g., miR-28, -31a, and −130a). These miRNAs, upon uptake by DRG neuron axons, suppress axon-stabilizing proteins DNA methyltransferase 3α, NUMB, SNAP-25, and GAP-43), directly contributing to axonal pathology (Jia et al., 2018). Thus, SC-derived EVs act as vectors for transmitting injury signals under diabetic conditions.

**FIGURE 1:**
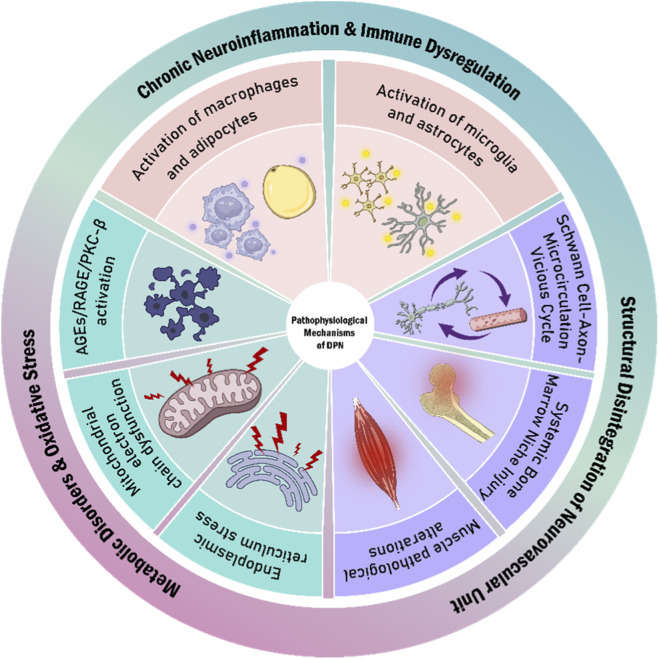
Schematic overview of core pathophysiological mechanisms of DPN. The pathogenesis of DPN involves a synergistic cascade of metabolic, immunological, and structural derangements. Metabolic disorders and oxidative stress are driven by AGEs/RAGE/PKC-β activation, mitochondrial electron transport chain dysfunction, and ER stress, collectively intensifying cellular oxidative damage. Chronic neuroinflammation and immune dysregulation are characterized by the activation of peripheral macrophages and adipocytes, alongside central microglia and astrocytes, promoting a pro-inflammatory microenvironment. Structural disintegration of neurovascular unit manifests as a vicious cycle of Schwann cell-axon-microcirculation impairment, coupled with systemic bone marrow niche injury and muscle pathological alterations. These interconnected axes drive the progressive neurodegeneration characteristic of the diabetic state.

Microcirculatory impairment leads to nerve ischemia. The resulting hypoxia damages neurons and SCs and dysregulates neurotrophic factor expression, creating a self-perpetuating cycle (Goldney et al., 2023; Group et al., 2022; Wang et al., 2025b). Diabetes also affects the bone marrow niche, where hematopoietic stem/progenitor cells acquire a pro-inflammatory secretory phenotype and can fuse with peripheral neurons *via* dysregulated insulin and TNF-α expression, directly inducing neuropathy (Katagi et al., 2021; Terashima et al., 2023). Muscle pathological changes follow denervation in DPN. Chronic denervation causes muscle fiber atrophy and degeneration, impaired satellite cell activation and differentiation, and accelerated functional decline. The neuro-inflammatory microenvironment, neurotrophic deficiency, and ischemia/hypoxia collectively cripple muscle regenerative potential, creating a cycle of muscle loss and functional impairment. These alterations, schematically illustrated in Figure 1, underscore the multi-tissue impact of DPN beyond the peripheral nervous system.

In summary, NVU disintegration is a multi-scale process driven by metabolic stress, oxidative damage, aberrant cell signaling, and EV-mediated intercellular communication (Figure 1). This interconnected network identifies clear therapeutic targets and supports multi-targeted repair strategies, such as stem cells and their EVs, which deliver a complex payload of molecules to correct diverse signaling defects.

Importantly, the molecular pathways discussed above do not operate in isolation; they feed into each other, forming a self-amplifying injury cycle. This interconnectedness explains why single-agent therapies targeting only one pathway have largely failed to reverse DPN. Effective disease modification will likely require multi-targeted strategies that address multiple nodes of this pathogenic network. Stem cell-derived EVs, with their naturally complex cargo of proteins and miRNAs, offer one such approach, as elaborated in the following sections.

## 3 Stem cell therapy for DPN: mechanisms, advances, and bottlenecks

### 3.1 Major stem cell types: sources and therapeutic potential

Stem cells, owing to their self-renewal capacity and multipotent differentiation potential, represent a promising therapeutic resource for DPN (Alizadeh et al., 2024). Differences in immunogenicity and differentiation propensity among stem cells from various sources confer distinct advantages and limitations in clinical application.

Mesenchymal stem cells (MSCs) have emerged as a major focus of translational research due to their abundant availability, potent immunomodulatory properties, and robust paracrine activity. MSCs can be obtained from various tissues including bone marrow, umbilical cord, placenta, adipose tissue, and dental pulp, exhibiting low immunogenicity and high expansion potential *in vitro*. Accumulating evidence indicates that MSCs ameliorate nerve dysfunction and structural damage in DPN primarily *via* secretion of a broad repertoire of growth factors and cytokines, which collectively promote neuroregeneration, angiogenesis, and inflammation resolution (Monfrini et al., 2017; Waterman et al., 2012). For instance, systemic administration of conditioned medium from human adipose-derived MSCs (ADSCs) significantly restores IENFD and improves thermal and mechanical sensitivity in diabetic mice (De Gregorio et al., 2020). Similarly, MSC-derived exosomes effectively enhance nerve blood flow perfusion and vascular density, alleviating neural ischemia and hypoxia. Furthermore, by inhibiting the TLR-4/NF-κB signaling pathway, they reduce inflammatory monocyte infiltration and suppress endothelial cell activation, creating a favorable microenvironment for neural repair (Fan et al., 2021; Lu et al., 2025). Clinically, a trial combining bone marrow-derived MSCs (BMSCs) with monocytes significantly reduced the incidence of DPN in type 2 diabetic patients (Wu et al., 2024).

Endothelial progenitor cells (EPCs) play a crucial role in promoting neovascularization in ischemic tissues and hold therapeutic promise for DPN. Preclinical studies show that local transplantation of bone marrow-derived EPCs into diabetic mice results in long-term engraftment within the sciatic nerve, particularly enriched around vasa nervorum, and markedly reverses hallmark deficits such as slowed NCV, decreased blood flow, and reduced capillary density (Jeong et al., 2009). Dental pulp stem cells (DPSCs), which can be harvested with minimal invasiveness and possess multilineage differentiation and immunomodulatory capacities, have shown notable potential in neural regeneration and diabetes-related complications (Paduano et al., 2021). Notably, cryopreserved human DPSCs, upon transplantation into diabetic animal models, significantly improve elevated sensory thresholds, impaired NCV, and reduced nerve perfusion, with functional benefits sustained long-term after a single administration (Hata et al., 2021; Omi et al., 2017). In chronic, long-term DPN models, DPSC transplantation promotes recovery of neurophysiology and neuropathology, including increased myelin thickness and area, as well as enhanced IENFD (Hata et al., 2020).

Among the 3 cell types discussed, MSCs have advanced the farthest in clinical translation. To date, MSCs are the only stem cell type that has entered human clinical trials for DPN, with preliminary evidence supporting their feasibility and safety (Alizadeh et al., 2024). A recent 8-year randomized controlled trial demonstrated that bone marrow MSC combination therapy significantly reduced DPN incidence compared with standard care (10.3% vs. 48.3%, *p* = 0.0015) (Wu et al., 2024), and a 2024 meta-analysis of seven controlled trials confirmed significant improvements in nerve conduction velocities and clinical symptom scores with an acceptable safety profile (Alizadeh et al., 2024). By contrast, studies on EPCs and DPSCs remain confined to preclinical animal models. This disparity likely reflects the relative ease of MSC isolation, their well-documented safety profile from other indications, and their robust paracrine activity that aligns with the current understanding of DPN pathogenesis. Existing clinical data further support the feasibility and efficacy of stem cell therapy for DPN. A meta-analysis revealed that stem cell therapies, primarily involving bone marrow-derived mononuclear cells (BM-MNCs) and umbilical cord-derived MSCs (UMSCs), significantly improved motor and sensory NCV, vibration perception threshold (VPT), and Toronto Clinical Scoring System (TCSS) scores, with adverse effects largely limited to transient injection-site pain and swelling (Alizadeh et al., 2024). The TCSS is a validated composite score that evaluates DPN severity based on symptom assessment (pain, numbness, paresthesia), deep tendon reflexes, and sensory testing (pinprick, temperature, light touch, vibration). Scores range from 0 to 19, with higher scores indicating more severe neuropathy; scores of 0–5 indicate no DPN, 6–8 mild, 9–11 moderate, and 12–19 severe DPN (Bril and Perkins, 2002). Originally developed and validated by Perkins *et al.* (Perkins et al., 2001), the TCSS has been widely employed as a clinical endpoint in DPN trials (Baszyńska-Wilk et al., 2026).

A detailed side-by-side comparison of stem cell sources, mechanisms, and clinical evidence is provided in Supplementary Table S1. These findings collectively underscore the favorable safety profile and clinical potential of stem cell-based approaches for DPN, while also highlighting the current gap between preclinical promise and clinical application for non-MSC cell types.

### 3.2 Core mechanisms of action: the shift to paracrine molecular signaling

In DPN therapy, the reparative effects of stem cells are now understood to be mediated predominantly through paracrine signaling, involving the release of a complex cocktail of bioactive factors and EVs, rather than direct cell replacement. This secretome mediates a coordinated, multimodal response at the molecular level, encompassing immunomodulation, angiogenesis, neurotrophic support, and microenvironmental remodeling (De Gregorio et al., 2020). The paracrine paradigm is supported by studies showing that systemic delivery of stem cell-conditioned medium, rich in secreted molecules, recapitulates the therapeutic benefits of whole-cell transplantation (De Gregorio et al., 2020).

Stem cells secrete a plethora of neurotrophic factors, including nerve growth factor (NGF), brain-derived neurotrophic factor (BDNF), glial cell line-derived neurotrophic factor (GDNF), and vascular endothelial growth factor (VEGF), that bind to receptors on neurons and SCs, activating pro-survival and pro-regenerative signaling cascades such as PI3K/Akt and MAPK/ERK (Evangelista et al., 2018; Smolinski et al., 2025; Sultan et al., 2021). A systematic review on growth factor signaling in peripheral nerve injury emphasizes that the therapeutic effects of these factors are mediated through their specific binding to cognate receptors, which then trigger downstream cascades (Li et al., 2020). NGF is essential for the survival and maintenance of peripheral sensory and sympathetic neurons, promoting axonal growth and supporting nociceptive function (Kim et al., 2025). BDNF acts on both sensory and motor neurons, enhancing synaptic plasticity, axonal regeneration, and neuronal survival under stress conditions. GDNF provides strong trophic support for motor neurons and dopaminergic neurons, and has been shown to promote motor axon regeneration and protect against motoneuron degeneration (Porcari et al., 2025). The coordinated activities of these factors are critical for peripheral nerve maintenance and repair. DPSC-CM, for instance, contains NGF, BDNF, and GDNF, which synergistically promote neurite outgrowth and extension (Sultan et al., 2021). Placenta-derived MSCs can activate SCs and modulate the Wnt signaling pathway to enhance remyelination and inhibit apoptosis (Pan et al., 2022).

Their immunomodulatory effects are achieved through the release of cytokines like interleukin-10 (IL-10) and transforming growth factor-β (TGF-β), as well as EVs carrying anti-inflammatory miRNAs. For example, MSC-derived EVs can modulate macrophage polarization by delivering miRNAs that target and inhibit the TLR4/NF-κB pathway, shifting the balance from a pro-inflammatory M1 to an anti-inflammatory M2 phenotype (Fan et al., 2021; Fan B. et al., 2020). In parallel, BMSCs protect SCs through multiple mechanisms. BMSC treatment downregulates pro-oxidant and ferroptosis-related proteins, reducing oxidative stress, iron accumulation, and ferroptosis in SCs, thereby preserving myelin integrity (He et al., 2026). Specifically, BMSC-derived factors suppress acyl-CoA synthetase long-chain family member 4 (ACSL4) expression (a key ferroptosis mediator) while upregulating glutathione peroxidase 4 (GPX4), restoring cellular antioxidant balance (Vahidinia et al., 2024; Xiao et al., 2026). Mechanistically, exosomal miR-219-5p targets ubiquitin-conjugating enzyme E2 Z (UBE2Z) to stabilize and activate NRF2, forming an anti-ferroptosis axis. Concurrently, MSC-EVs inhibit c-Jun N-terminal kinase (JNK) phosphorylation to block Kelch-like ECH-associated protein 1 (KEAP1)-mediated NRF2 degradation, promoting NRF2 nuclear translocation and activating downstream antioxidant gene expression (Wu J. et al., 2025). These coordinated actions collectively mitigate mitochondrial dysfunction and lipid peroxidation in SCs under diabetic conditions.

In promoting angiogenesis, transplanted stem cells home to the neurovascular niche, upregulating angiogenic gene expression and releasing factors like VEGF that act on endothelial cells to stimulate vessel formation (Han et al., 2016; Lehmann and Hoke, 2016; Mizukami and Yagihashi, 2016). For example, injected BMSCs localize near vasa nervorum, upregulate angiogenic gene expression, markedly increase endoneurial vascular density, and improve ultrastructural integrity of myelinated fibers (Han et al., 2016; Lehmann and Hoke, 2016). Bone marrow-derived EPCs similarly migrate and incorporate into perineural vessel walls, directly augmenting local angiogenesis (Mizukami and Yagihashi, 2016). Furthermore, transplantation of iPSC-derived neural crest-like cells into the hind limbs of diabetic mice enhances neurotrophic factor expression, improves sensory dysfunction, and increases muscle capillary density, highlighting systemic circulatory support (Mizukami and Yagihashi, 2016).

Stem cell therapy can also restore the impaired endogenous repair capacity. Diabetes-induced downregulation of stromal cell-derived factor-1α (SDF-1α) in injured tissues compromises mobilization and homing of repair cells like EPCs (Kim et al., 2013). Combined use of C-X-C motif chemokine receptor 4 (CXCR4) antagonists AMD3100 and local SDF-1α supplementation effectively mobilizes and recruits bone marrow-derived repair cells such as CD133^+^/CD34^+^ EPCs and M2 macrophages to lesion sites, thereby improving NCV and vascular structure (Qi et al., 2020). In case of more severe mobilization defects, co-administration of granulocyte colony-stimulating factor (G-CSF) and plerixafor to mobilize autologous BM-MNCs, or hydrogen sulfide-based interventions to rescue cell function, offer novel avenues for refractory DPN (Mao et al., 2019; Santopaolo et al., 2020).

Translating these preclinical findings into clinical practice faces several challenges. First, species differences in nerve regeneration capacity, rodents regenerate faster and more completely than humans, which mean that promising results in animal models may not directly translate to patients (Marsh et al., 2024). Second, dosing optimization remains a hurdle: effective preclinical doses often do not scale linearly to human-equivalent doses due to differences in body size, metabolism, and immune responses (Naota et al., 2025; Ye et al., 2025). Third, long-term safety monitoring is essential, as potential tumorigenic risk or late adverse events may take years or decades to manifest, which is difficult to capture in animal studies with short follow-up periods (Leonov et al., 2025). These translational gaps highlight the need for careful dose-finding studies, long-term follow-up registries, and the development of robust biomarkers to predict clinical response before large-scale human trials (Zhao et al., 2025).

**FIGURE 2:**
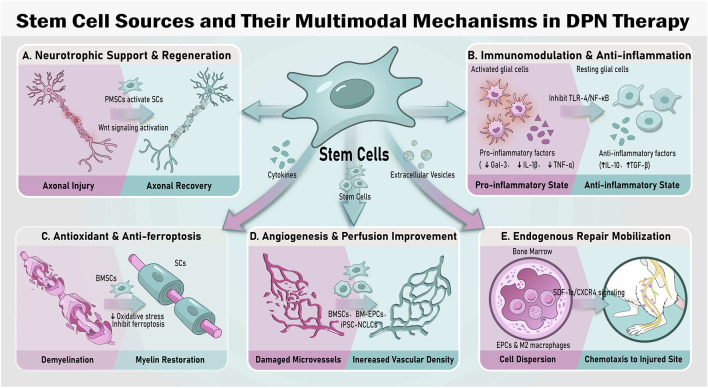
Stem cell sources and their multimodal mechanisms in DPN therapy. Stem cells and their derivatives (cytokines, EVs) exert neuroprotective effects through five primary axes: **(A)** Neurotrophic support and regeneration: activation of Wnt signaling facilitates the transition from axonal injury to recovery; **(B)** Immunomodulation and anti-inflammation: suppression of the TLR-4/NF-κB pathway shifts the microenvironment from a pro-inflammatory state (galectin-3, IL-1β, TNF-α) to an anti-inflammatory state (IL-10, TGF-β), accompanied by the stabilization of glial cells; **(C)** Antioxidant and anti-ferroptosis: reduction of oxidative stress and inhibition of ferroptosis promote myelin restoration following demyelination; **(D)** Angiogenesis and perfusion improvement: mobilization of BMSCs, BM-EPCs, and induced pluripotent stem cells (iPSCs)-NCLCs restores vascular density in damaged microvessels; **(E)** Endogenous repair mobilization: stimulation of the SDF-1α/CXCR4 signaling axis promotes the dispersion and chemotaxis of EPCs and M2 macrophages from the bone marrow to injured neural sites.

In summary, the therapeutic mechanism of stem cells in DPN is a multidimensional, networked repair process dominated by paracrine effects. Stem cells from different sources, through their secretome, particularly EVs as natural nanocarriers, act synergistically across key pathological axes including immunomodulation, neurotrophic support, angiogenesis, and endogenous cell recruitment, collectively reconstructing a microenvironment conductive to nerve regeneration (Figure 2; Supplementary Table S1). This insight not only underpins the paradigm shift from cell-based to cell-free therapeutics but also provides a specific roadmap: improving cell survival and homing efficiency through preconditioning, optimizing EV standardization for consistent molecular payloads, and developing combination therapies that target multiple pathological axes simultaneously.

### 3.3 Clinical translation of stem cell therapy for DPN: challenges and optimization strategies

Despite promising potential of stem cells in DPN treatment, their clinical translation faces multiple challenges. Low cell survival, poor persistence, and insufficient homing efficiency are key factors limiting efficacy. Studies show that BMSCs exhibit limited migration ability in diabetic models, hindering precise targeting of injured nerves. Genetic modification, such as overexpression of Neuritin to activate the SDF-1α/CXCR4-PI3K/Akt axis, can partially improve migration, but this introduces new complexities and safety concerns (Zhang et al., 2022). They include insertional mutagenesis from viral vectors, which may disrupt tumor suppressor genes or activate proto-oncogenes; long-term overexpression-associated tumorigenic risk, with evidence that extended passaging of genetically modified MSCs can lead to chromosomal abnormalities and malignant transformation; off-target effects, particularly when using CRISPR/Cas9 or similar editing tools; ethical concerns regarding permanent genetic alterations in therapeutic cells, including informed consent and potential germline transmission; and a lack of long-term safety data, as most preclinical studies have short follow-up periods that may not capture late-onset adverse events (Dashti et al., 2025; Jeong et al., 2011; Liu et al., 2025; Ortiz-Bueno et al., 2026; Patel et al., 2025). Furthermore, substantial heterogeneity in stem cell products, stemming from variations in tissue source, donor characteristics, isolation protocols, and culture conditions, undermines reproducibility and standardization. Thus, establishing internationally harmonized guidelines for cell manufacturing, characterization, and functional validation is urgently needed.

On the safety front, although short-term safety appears acceptable (Alizadeh et al., 2024), immunogenicity and long-term tumorigenic risks warrant rigorous assessment, particularly for genetically modified cells. Long-term surveillance and advanced *in vivo* tracking technologies are therefore essential. Critically, there is a lack of objective biomarkers to assess clinical efficacy. Current endpoints, such as subjective symptom scores and electrophysiological measures like NCV, lack sensitivity to small-fiber or microstructural changes and often poorly reflect underlying molecular or structural improvements. Development of robust, multimodal assessment systems based on neuroproteins, inflammatory factor profiles, and advanced neuroimaging is imperative.

Notably, mounting evidence indicates that the therapeutic benefits of MSCs are largely mediated by their secretome (De Gregorio et al., 2020), strongly supporting the development of cell-free alternatives. To enhance efficacy, *in vitro* pretreatment of MSCs (e.g., simulating hypoxia, using deferoxamine) can significantly upregulate their paracrine potency, thereby augmenting reparative capacity (Oses et al., 2017). Nevertheless, clinical reality presents sobering challenges: a recent Phase II trial found that intramuscular injection of an MSC product in DPN patients did not yield statistically significant improvements in IENFD (Gibbons et al., 2021), highlighting the formidable obstacle of achieving meaningful structural nerve regeneration in humans. Meanwhile, combinatorial approaches show promise. For instance, co-transplantation of MSCs with pancreatic islets not only reduces islet dosage but also synergistically alleviates neuropathy symptoms, enhancing the feasibility of integrated metabolic-neurological therapy (Monfrini et al., 2017).

Collectively, the clinical translation of stem cell therapy for DPN requires systematically addressing multiple challenges including poor cell survival and homing, product heterogeneity, safety uncertainties, and inadequate efficacy assessment. Future progress hinges on deepening mechanistic understanding of paracrine actions, advancing cell-free strategies, optimizing preconditioning and delivery protocols, developing objective assessment systems, and designing effective combination strategies. These efforts will pave the way toward mature, reliable, and widely applicable stem cell-inspired therapies for DPN.

## 4 EVs: the cell-free key to unlocking the therapeutic potential of stem cells

### 4.1 Biological characteristics, sources, and therapeutic advantages of EVs

As introduced earlier, EVs are lipid bilayer-enclosed particles that mediate intercellular communication. A recent review on peripheral nerve regeneration highlights that sEVs are key regulators of this intercellular crosstalk, particularly among neurons, SCs, macrophages, and fibroblasts (Izhiman and Esfandiari, 2024). Their molecular cargo, including proteins, lipids, and nucleic acids (e.g., mRNA, miRNA, mitochondrial DNA), is highly dependent on the parent cell type and its physiological state (Chandrasekera and Katare, 2022; Raghav et al., 2021). For instance, exosomes are characteristically enriched in tetraspanins (CD9, CD63, CD81) and heat shock proteins. Specific integrins and adhesion molecules on their surface determine cellular tropism, while the lipid bilayer protects the cargo, ensuring efficient delivery of molecular signals to target cells (Izquierdo-Altarejos et al., 2024; Kalluri and LeBleu, 2020; Raghav et al., 2021; Zeng et al., 2022).

From a clinical application perspective, stem cell-derived EVs offer several distinct advantages over conventional drug delivery systems and direct stem cell transplantation. First, they lack intact cellular structures, thereby avoiding the risks of ectopic tissue formation and tumorigenesis associated with cell therapy, and exhibit markedly lower immunogenicity, enabling effective evasion of immune rejection (Wu T. et al., 2025). Second, EVs can cross biological barriers efficiently. Their nanoscale size and lipid bilayer structure allow them to traverse the blood-brain barrier and blood-nerve barrier, delivering functional biomolecules to damaged neural tissues, a critical prerequisite for treating peripheral neuropathy (Krishnan et al., 2024). Third, EVs can be engineered for precise targeted delivery. Through genetic or chemical engineering, EVs can display specific targeting ligands on their membrane surfaces, enabling “molecular navigation” toward highly expressed receptors in diseased tissues and ensuring accurate enrichment at injury sites (Shie et al., 2026). Fourth, EVs are inherently stable and amenable to bioengineering; they can be modified to enhance their intrinsic molecular cargo or to carry exogenous therapeutic molecules, making them highly versatile platforms for targeted molecular therapy (Chen et al., 2025; Wang et al., 2025c; Yang et al., 2024).

Despite these advantages, EV-based therapies face several translational challenges that must be addressed before widespread clinical application. First, their short biological half-life in circulation (typically minutes to hours) limits systemic delivery efficiency, and biodistribution patterns vary significantly depending on administration route, EV source, and surface properties (Zeng et al., 2022). Second, standardizing EV production and dosing is complicated by heterogeneity in isolation methods (ultracentrifugation, precipitation, size-exclusion chromatography) and quantification metrics (particle number, protein content, or RNA amount), leading to poor comparability across studies (Bharti et al., 2025). Third, scalable Good Manufacturing Practice (GMP)-compliant production remains a technical bottleneck; current manufacturing yields are often insufficient for clinical doses, and batch-to-batch consistency is difficult to achieve (Zhang et al., 2026). Fourth, the selection of the most appropriate route of administration (e.g., intravenous, local, or intrathecal) requires systematic optimization for DPN-specific applications, as each route presents distinct pharmacokinetic and safety profiles (Huang et al., 2025). Fifth, long-term safety data, particularly for engineered EVs carrying exogenous cargo, are still limited, and potential off-target effects or immunogenicity of modified EV surfaces need further investigation (Balaraman et al., 2025).

Thus, a balanced understanding of these strengths and limitations is essential for guiding the rational development of EV-based therapies for DPN.

### 4.2 Multidimensional repair mechanisms of EVs in DPN: a focus on molecular pathways

#### 4.2.1 Alleviation of metabolic dysregulation and antioxidant stress protection *via* specific molecular pathways

Under diabetic conditions, EVs reduce metabolic disturbances and alleviate oxidative injury by modulating specific molecular pathways. MSC-EVs can improve insulin sensitivity by activating the PI3K/Akt signaling pathway in target cells, thereby positively regulating insulin receptor substrate (IRS) phosphorylation (Li et al., 2022). In terms of antioxidant defense, EVs exert significant neuroprotective effects by delivering key molecular activators. For example, exosomes derived from BMSCs overexpressing the deacetylase sirtuin 1 (Exo-SIRT1) mitigate hyperglycemia-induced oxidative stress in DPN models by activating the NRF2/HO-1 antioxidant pathway in recipient cells (Shan et al., 2022). Similarly, ADSC-EVs carrying miR-130a-3p enhance antioxidant enzyme activity and reduce oxidative damage by activating the NRF2/hypoxia-inducible factor 1-α (HIF1α)/ACTA1 axis (Chen et al., 2023). Furthermore, SC-derived exosomes preconditioned with paeoniflorin protect DRG neurons from apoptosis by modulating the ER stress-related IRE1α pathway (Zhu et al., 2021). Collectively, these findings demonstrate that EVs mitigate metabolic dysregulation and oxidative stress by activating NRF2-driven antioxidant programs and modulating ER stress responses, thereby protecting neurons and SCs from hyperglycemia-induced damage.

#### 4.2.2 Immune reprogramming and anti-inflammatory effects through modulation of inflammatory signaling

EVs actively reshape the immune microenvironment during DPN progression by serving as key carriers of anti-inflammatory molecular signals. MSC-Exos deliver specific miRNAs (e.g., let-7a, miR-23a, miR-125b, miR-146a) that cooperatively target and inhibit the TLR4/NF-κB signaling cascade in macrophages and endothelial cells, leading to reduced expression of pro-inflammatory cytokines and adhesion molecules including intercellular adhesion molecule-1 (ICAM-1) and vascular cell adhesion molecule-1 (VCAM-1) (Fan et al., 2021; Fan B. et al., 2020). This molecular intervention promotes macrophage polarization from the M1 to the M2 phenotype. Engineering strategies can amplify this effect; for instance, MSC-EVs enriched with miR-146a display enhanced anti-inflammatory capacity and preferentially accumulate in neural tissues, leading to significant DPN amelioration (Fan et al., 2021). EVs from other sources, such as NSC-EVs loaded with the anti-inflammatory agent sinomenine (SIN, NSC-EVs@SIN), specifically target microglia and inhibit the WNT5a/TRPV1 signaling pathway, driving their polarization towards the M2 phenotype (Chen et al., 2025). SC-EVs enriched in miR-214 similarly reduce macrophage infiltration and inhibit the TLR4/NF-κB cascade (Wang L. et al., 2025). Defining these specific miRNA cargos and their target pathways is not merely descriptive; it directly informs the design of engineered EVs with enhanced anti-inflammatory potency for DPN. For example, enriching EVs with miR-214 or targeting WNT5a/TRPV1 could yield more effective cell-free therapies that precisely modulate neuroinflammation while avoiding the side effects of systemic immunosuppression.

#### 4.2.3 Neuro-vascular coordinated repair *via* delivery of pro-angiogenic and neurotrophic signals

EVs facilitate multi-target repair of the damaged neurovascular unit by synergistically promoting angiogenesis and nerve regeneration through the delivery of complementary molecular signals. In stimulating vascularization, ADSC-EVs, *via* their highly expressed miR-130a-3p, downregulate DNA methyltransferase 1 (DNMT1) and activate the NRF2/HIF1α/ACTA1 axis, effectively enhancing angiogenesis (Chen et al., 2023). MSC-Exos directly improve endothelial cell function by delivering growth factors like fibroblast growth factor (FGF), VEGFA, and NGF, which bind to receptors on endothelial cells and activate pro-angiogenic pathways (Fan B. et al., 2020). Pericyte-derived nanovesicles rely on lipocalin 2 (Lcn2) to activate PI3K/Akt and MAPK pathways in vascular and nerve cells, enhancing regeneration (Anita et al., 2022). Regarding neural restoration, EVs derived from healthy SCs increase IENFD and promote remyelination. Their miRNA cargo, including miR-21, miR-27a, and miR-146a, promotes axonal growth and myelination by downregulating regeneration-blocking proteins like phosphatase and tensin homolog (PTEN), JNK, and semaphorin 6A (SEMA6A), thereby fostering a pro-regenerative molecular environment (Liu et al., 2022; Wang et al., 2020).

**FIGURE 3:**
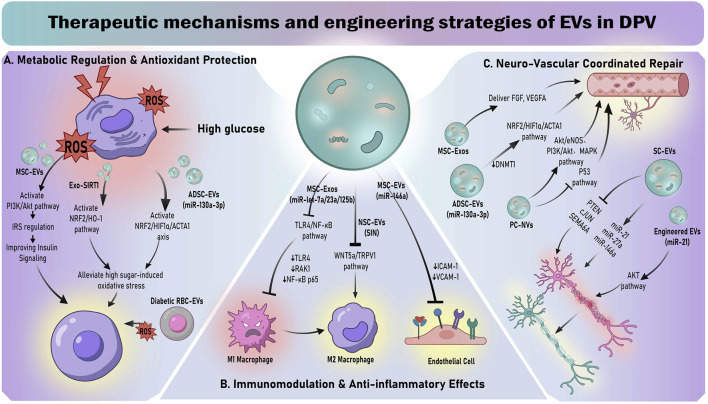
Therapeutic mechanisms and engineering strategies of EVs in DPN. EVs serve as critical paracrine mediators for coordinated tissue repair through three primary functional domains: **(A)** Metabolic regulation and antioxidant protection: engineered and native EVs (e.g., Exo-SIRT1, ADSC-EVs) alleviate high-glucose-induced oxidative stress by activating the PI3K/Akt and NRF2/HO-1 pathways, improving insulin signaling and modulating ER stress; **(B)** Immunomodulation and anti-inflammatory effects: EVs promote M1-to-M2 macrophage polarization and downregulate endothelial adhesion molecules (ICAM-1, VCAM-1) by targeting TLR4/NF-κB, WNT5a/TRPV1, and miRNA-mediated signaling; **(C)** Neuro-vascular coordinated repair: EVs deliver specific cargos (e.g., FGF, VEGFA, miRNAs) to synergistically promote angiogenesis and axonal regeneration. This coordinated repair is mediated by the activation of Akt/endothelial nitric oxide synthase (eNOS), PI3K/Akt, and MAPK pathways, alongside the inhibition of pro-degenerative signals like PTEN and tumor protein p53.

Together, these findings underscore the capacity of stem cell-derived EVs to achieve multisite repair of the neurovascular unit by delivering a coordinated payload of molecular signals. By enhancing angiogenesis to provide trophic support while directly acting on SCs and neurons to promote structural regeneration *via* activation of pathways like PI3K/Akt, MAPK, and NRF2/HO-1, EVs enable a multi-targeted molecular intervention across the core pathological axes of DPN (Figure 3; Supplementary Table S2). Thus, the coordinated delivery of pro-angiogenic and neurotrophic signals by EVs addresses both the vascular and neural components of NVU dysfunction, offering an integrated strategy for neurovascular repair.

### 4.3 Engineering and delivery technology innovation for EVs: enhancing molecular cargo and targeting

To overcome inherent limitations of native EVs, such as insufficient targeting specificity and low therapeutic potency, engineering modifications have become critical. These innovations aim to enhance the EV’s molecular cargo or to improve its delivery to specific cell types. In DPN-specific research, EV engineering strategies have focused on enhancing the delivery of neuroprotective and anti-inflammatory cargo, such as miR-146a, miR-214, and SIRT1, to damaged peripheral nerves, while general EV engineering platforms have explored broader applications in cancer immunotherapy and tissue regeneration.

Endogenous modification involves preconditioning parent cells or employing genetic manipulation to enhance the EV’s intrinsic molecular payload. For example, engineering SCs to overexpress miR-214 leads to the secretion of EVs enriched with this anti-inflammatory and pro-regenerative miRNA (Wang L. et al., 2025). Similarly, loading parent cells with a drug like SIN results in NSC-EVs@SIN, which carry this therapeutic molecule and target microglia (Chen et al., 2025).

Exogenous modification focuses on surface engineering of isolated EVs to introduce targeting ligands that improve homing to specific cells. For instance, conjugating EVs with the RVG peptide enables them to target neuronal acetylcholine receptors. A more sophisticated system involves modifying the ZH-1c aptamer onto NSC-EVs@SIN, creating ZH-1c-EVs@SIN, which achieves highly efficient, targeted drug delivery to microglia (Chen et al., 2025). Surface modification with the photoreceptor targeting peptide MH42 significantly enhances EV accumulation at sites of diabetic retinopathy (Sun et al., 2025).

Advanced delivery platforms, such as biomaterial scaffolds, aim to extend local EV retention, enabling sustained release of their molecular cargo. For example, integrating BMSC-EVs into a conductive rGO-GelMA-PCL conduit enhances vascularization and axonal sprouting in nerve defect models (Zhang et al., 2023). An innovative hybrid platform fuses BMSC-Exos with polypyrrole nanoparticle-loaded liposomes, creating a system that delivers combined biochemical and electrical stimulation for comprehensive DPN treatment (Singh et al., 2021).

Together, these engineering and delivery innovations are overcoming therapeutic bottlenecks by optimizing the molecular cargo of EVs, refining their targeting for precise molecular delivery, and ensuring durable retention for sustained molecular action. The clinical relevance of these engineering strategies is clear: without improved targeting and retention, even the most potent EV cargo will fail to reach diseased nerves in sufficient quantities to reverse established pathology. Advancing these technologies from bench to bedside is therefore a prerequisite for translating EV-based therapies into effective DPN treatments.

## 5 Clinical translation of stem cell and EVs therapy for DPN: current status, challenges, and pathways

### 5.1 Current status and limitations of preclinical research

Current preclinical research on stem cell and EVs therapy for DPN predominantly relies on animal models, including streptozotocin (STZ)-induced diabetes, high-fat diet models, and *db/db* mice. Studies demonstrate that BMSCs significantly promote neurological functional recovery in diabetic rats, improve NCV, and facilitate axonal regeneration and angiogenesis, primarily through activation of the glycogen synthase kinase-3β (GSK-3β)/β-catenin signaling pathway (He et al., 2020; Mohamed et al., 2024). Meanwhile, stem cell-derived EVs, particularly SC-EVs, exert neuroprotective and anti-inflammatory effects by delivering specific miRNAs such as miR-214 (Wang L. et al., 2025). ADSC-EVs ameliorate DPN pathology by transferring molecules like miR-130a-3p to promote SC proliferation and inhibit apoptosis (Chen et al., 2023). These studies elucidate the multifaceted mechanisms by which stem cells and EVs promote vascular regeneration, remyelination, and immunomodulation (Ahmed and Al-Massri, 2024; Chen et al., 2025).

The route of administration significantly influences therapeutic efficacy and mechanistic outcomes. Local injection enables direct targeting of injured nerves and facilitates localized microenvironmental repair. Systemic intravenous delivery may act primarily through modulation of systemic inflammation and immune responses (Alizadeh et al., 2024). Intrathecal injection, acting directly on the central nervous system periphery, possesses unique advantages in alleviating neuroinflammation and promoting nerve regeneration. However, high quality comparative studies evaluating the relative efficacy of these delivery routes are currently lacking, and the long-term safety profiles of each approach remain to be fully established.

Despite their utility, existing animal models exhibit notable limitations. The STZ-induced model primarily recapitulates acute pancreatic β cell damage in type 1 diabetes, whereas *db/db* mice model the obesity and insulin resistance features of type 2 diabetes; neither fully mirrors the multifactorial etiology and chronic, progressive neural degeneration observed in human DPN (Lee, 2026). Furthermore, diabetic patients often present complex metabolic abnormalities, microangiopathy, and neuroimmune pathologies that are incompletely modeled in animal models, thereby limiting the translational relevance of preclinical data. Additionally, the neurological function assessment metrics in animal models differ from clinical evaluations, coupled with variability across studies in stem cell source, dosage, administration frequency, and timing of intervention, which further complicates data interpretation and generalizability.

Overall, preclinical research has demonstrated the potential of stem cells and EVs to promote neuroprotection, structural repair, and functional recovery in DPN, revealing diverse mechanisms and delivery modalities. The inherent limitations of current animal models and the heterogeneity in experimental design constitute major barriers to clinical translation, particularly for validating the specific molecular mechanisms of action identified *in vitro*.

### 5.2 Core challenges in clinical translation

#### 5.2.1 Safety challenges associated with stem cell therapy

While stem cell therapy holds promise for DPN, clinical application faces several safety and functional hurdles. Poor post-transplantation cell viability remains a key limitation. Engrafted cells undergo rapid clearance or apoptosis in the diabetic microenvironment, often within days, due to oxidative stress, inflammation, and potential host immune responses (Karimova et al., 2022). Although MSCs have immunomodulatory properties, allogeneic transplantation still carries a risk of immune rejection.

Tumorigenic potential is another concern. The self-renewal capacity of stem cells, if dysregulated, may lead to aberrant growth. For example, MSCs subjected to extensive *in vitro* passaging can acquire chromosomal abnormalities, increasing oncogenic risk (Jeong et al., 2011). iPSCs pose a greater threat when differentiation is incomplete, as residual undifferentiated cells may form teratomas (Mashima et al., 2021). In diabetic patients, especially those with type 2 diabetes, endogenous EPCs show functional deficits, including reduced numbers, impaired migration, adhesion, and tubulogenesis. The diabetic systemic environment not only weakens endogenous repair but also limits transplanted or genetically modified EPC efficacy (Jiang et al., 2021; Yiu and Tse, 2014).

Beyond biological risks, stem cell therapy confronts ethical and technical hurdles. The use of embryonic stem cell (ESC) remains ethically controversial due to the required destruction of human embryos, raising fundamental moral and legal questions regarding the status of pre-implantation embryos (Writing Group of the et al., 2024). iPSCs avoid this concern by enabling patient-specific cells without embryo destruction, but they introduce complex preparation procedures and potential genetic instability, including copy number variations and point mutations from reprogramming and long-term culture (Edwards et al., 2024; Williams et al., 2025). Technical standardization of stem cell isolation, expansion, purification, and preservation is essential; deviations compromise product quality and therapeutic consistency (Liu et al., 2023). Robust *in vivo* cell tracking and long-term functional monitoring methods remain underdeveloped (Cen et al., 2016; Cen et al., 2026). Clinical trials have documented risks such as immune reactions, infections, and route-related complications (Hering et al., 2025; Marei, 2025).

#### 5.2.2 Translational bottlenecks for stem cell and EV-based therapies

Current translational efforts face several quantifiable bottlenecks. First, transplanted stem cells encounter a hostile lesion microenvironment, including high glucose, oxidative stress, and inflammation, leading to >90% apoptosis within 72 h and loss of paracrine function exceeding 50% (Ahmed and Al-Massri, 2024). Second, paracrine factor expression in stem cells varies up to three-fold across donors and batches, causing poor reproducibility of clinical outcomes (Alizadeh et al., 2024). Third, systemic delivery efficiency is extremely low. Most intravenously infused stem cells are trapped in the lungs or cleared by the immune system, with <0.1% reaching neural tissue. Native EVs have a circulation half-life of only 30–60 min; >90% are rapidly cleared by the liver and spleen, resulting in <1% accumulation in target nerves (Lu et al., 2025). Fourth, conventional 2D culture is difficult to scale, and cells tend to senesce and lose stemness during expansion, failing to meet clinical demand (Hodge et al., 2025; Wong et al., 2023). Fifth, EV preparation lacks standardization. Ultracentrifugation yields only 1–10 μg vesicles per 10^6^ stem cells, far below milligram-level clinical doses. Different isolation methods produce vesicles with vastly different purity and residual impurities, making dose standardization impossible. Native EVs also lack neural tropism, limiting local effective concentrations (Tajabadi et al., 2025).

These bottlenecks must be systematically addressed before stem cell or EV therapies can be reliably translated. Compared to stem cells, EVs offer a better safety profile, but production standardization remains the foremost challenge. Large-scale, GMP-compliant production must ensure consistency and potency of the EV molecular cargo. Adherence to Minimal Information for Studies of EV (MISEV) guidelines and techniques such as NTA, TEM, and Western blotting are essential for characterization by size, morphology, and surface markers (e.g., CD9, CD63, CD81) (Vaiaki and Falasca, 2024). However, the absence of internationally harmonized quality benchmarks for EV molecular composition (e.g., specific miRNA or protein content) hinders cross-study comparability and clinical reproducibility. Pharmacokinetic profiling and long-term safety assessment, especially for engineered EVs with modified molecular cargo, need further investigation. Clinical trial design should incorporate robust endpoints that reflect molecular and structural improvements, moving beyond subjective symptom scores.

### 5.3 Future prospects and frontier directions: precision molecular therapeutics

Moving forward, stem cell and EV therapies will advance through strategy optimization and technological innovation towards precise molecular intervention.

Combination and personalized approaches. Given the multifactorial pathogenesis of DPN, combining engineered EVs with different molecular payloads (e.g., anti-inflammatory miRNAs and pro-angiogenic proteins) may yield better outcomes than single-agent strategies (Chen et al., 2025). Patient-tailored selection based on molecular phenotyping (e.g., dominant inflammatory *versus* vascular insufficiency profile) could match each patient with the most relevant EV product.

Emerging technologies with balanced risks. Gene editing (e.g., CRISPR/Cas9) can engineer parent stem cells to stably modify the molecular cargo of secreted EVs, amplifying reparative functions (Wang L. et al., 2025). However, CRISPR/Cas9 carries risks: off-target genomic modifications, potential immunogenicity of Cas9 protein, and ethical concerns about germline editing (intergenerational consent, equity, and genetic enhancement beyond therapy). AI and big data analytics may help predict optimal EV cargo compositions and design engineering strategies, but they also pose challenges: patient data privacy, algorithmic bias that may not generalize across populations, and the “black box” nature of deep learning models, which limits interpretability in clinical decisions and raises accountability concerns. Therefore, rigorous validation, transparent reporting, and regulatory oversight are essential before clinical integration.

Regulatory and industrial pathway. Comprehensive regulatory guidelines for EV-based biologics are urgently needed, covering the entire value chain from manufacturing to clinical validation. Industrial chain integration, cost reduction through scaled-up production, and high-quality evidence from well-designed multicenter trials will accelerate clinical translation and benefit DPN patients.

## 6 Conclusion

This review has systematically delineated the complex molecular landscape of DPN pathophysiology, highlighting the interconnected signaling pathways of metabolic stress, neuroinflammation, and neurovascular dysfunction. We have provided an in-depth analysis of the molecular mechanisms by which stem cells and, more importantly, their secreted EVs, orchestrate neural repair. The therapeutic shift from whole-cell therapies to EV-based cell-free strategies represents a logical progression towards more defined, targeted, and controllable interventions. EVs function as nature’s own multi-molecular drug delivery systems, capable of simultaneously modulating several dysregulated pathways in DPN by delivering a synergistic payload of proteins and miRNAs.

By focusing on the molecular cargo of EVs, such as miR-146a for immunomodulation, miR-130a-3p for antioxidant and angiogenic effects, and SIRT1 protein for metabolic regulation, we can begin to understand their therapeutic effects not as a single action, but as a coordinated multi-target intervention at the molecular level. This understanding matters because it shifts the therapeutic paradigm from seeking a single “magic bullet” to embracing combination strategies that address the full complexity of DPN pathogenesis. It also provides a rational framework for patient stratification: individuals with predominantly inflammatory profiles may benefit more from miR-146a-enriched EVs, while those with vascular insufficiency may require pro-angiogenic cargo such as miR-130a-3p. This perspective highlights the immense potential for bioengineering to enhance these natural properties, creating next-generation EV therapeutics with optimized molecular payloads and refined targeting capabilities for precision medicine in DPN.

Looking ahead, interdisciplinary collaboration will be essential for overcoming translational hurdles. The integration of systems biology, bioengineering, and clinical medicine holds great promise for developing next-generation EV therapeutics. As these technologies mature, EV-based therapies are poised to emerge as a transformative and molecularly precise strategy for DPN.

## Glossary

ACSL4: Acyl-CoA synthetase long-chain family member 4
ADSC: Adipose-derived mesenchymal stem cell
AGE: Advanced glycation end-product
AI: Artificial intelligence
BDNF: Brain-derived neurotrophic factor
BM-MNC: Bone marrow-derived mononuclear cell
BMSC: Bone marrow-derived mesenchymal stem cell
CXCR4: C-X-C motif chemokine receptor 4
DNMT1: DNA methyltransferase 1
DPN: Diabetic peripheral neuropathy
DPSC: Dental pulp stem cell
DRG: Dorsal root ganglion
eNOS: Endothelial nitric oxide synthase
EPC: Endothelial progenitor cell
ER: Endoplasmic reticulum
ESC: Embryonic stem cell
EV: Extracellular vesicle
Exo: Exosome
FGF: Fibroblast growth factor
G-CSF: Granulocyte colony-stimulating factor
GDNF: Glial cell line-derived neurotrophic factor
GMP: Good Manufacturing Practice
GPX4: Glutathione peroxidase 4
GSH: Glutathione
GSK-3β: Glycogen synthase kinase-3β
HIF1α: Hypoxia-inducible factor 1-α
HO-1: Heme oxygenase 1
ICAM-1: Intercellular adhesion molecule-1
IENFD: Intraepidermal nerve fiber density
IL: Interleukin
iPSC: Induced pluripotent stem cell
IRE1α-CHOP: Inositol-requiring enzyme 1α-C/EBP homologous protein
IRS: Insulin receptor substrate
JNK: c-Jun N-terminal kinase
KEAP1: Kelch-like ECH-associated protein 1
Lcn2: Lipocalin 2
MAPK: Mitogen-activated protein kinase
MDA: Malondialdehyde
miRNAs: microRNAs
MISEV: Minimal Information for Studies of Extracellular Vesicles
MNSI: Michigan Neuropathy Screening Instrument
MRI: Magnetic resonance imaging
MSC: Mesenchymal stem cell
**NAD** ^**+**^: Nicotinamide adenine dinucleotide
NADPH: Reduced Nicotinamide Adenine Dinucleotide Phosphate
NCV: Nerve conduction velocity
NF-κB: Nuclear factor kappa-light-chain-enhancer of activated B cells
NGF: Nerve growth factor
NO: Nitric oxide
NRF2: Nuclear factor erythroid 2-related factor 2
NSC: Neural stem cell
NTA: Nanoparticle tracking analysis
NVU: Neurovascular unit
PI3K/Akt: Phosphoinositide 3-kinase/AKT serine/threonine kinase
PKC: Protein kinase C
PTEN: Phosphatase and tensin homolog
RAGE: Receptor for advanced glycation end-products
RNS: Reactive nitrogen species
ROS: Reactive oxygen species
SARM1: Sterile alpha and TIR motif containing 1
SC: Schwann cell
SDF-1α: Stromal cell-derived factor-1α
SEMA6A: Semaphorin 6A
sEVs: Small extracellular vesicles
SIN: Sinomenine
Sirt3: Sirtuin 3
SOD: Superoxide dismutase
STZ: Streptozotocin
TCSS: Toronto Clinical Scoring System
TEM: Transmission electron microscopy
TGF-β: Transforming growth factor-β
TLR: Toll-like receptor
TNF-α: Tumor necrosis factor-α
UBE2Z: Ubiquitin-conjugating enzyme E2 Z
UMSC: Umbilical cord-derived mesenchymal stem cell
VCAM-1: Vascular cell adhesion molecule-1
VEGF: Vascular endothelial growth factor
VPT: Vibration perception threshold
WNT5a/TRPV1: Wingless-type MMTV integration site family, member 5a/Transient receptor potential vanilloid 1
