# Supplementary material for: From pathophysiology to therapy: molecular mechanisms of stem cell and extracellular vesicle-mediated repair in diabetic peripheral neuropathy
Source: Front Cell Dev Biol. 2026 Jun 16;14:1854350. doi: 10.3389/fcell.2026.1854350 (PMC13314850; doi:10.3389/fcell.2026.1854350)
Supplement: Supplementary file 1 [file Table1.docx]

**Table S1. Therapeutic effects and mechanisms of stem cell therapies in DPN.**

| **Cell Origin** | **Product Type** | **Dose & Regimen** | **Disease Model** | ***In Vitro* Effects** | ***In Vivo* Outcomes** | **Proposed Mechanism** | **Ref.** |
| --- | --- | --- | --- | --- | --- | --- | --- |
| MSCs | Cells | Microencapsulated islets (2000/rat) and 10^6^ MSCs/rat | STZ-induced (60 mg/kg, i.p.), male Lewis rats (8 wk) | Not specified | Reduced islet requirement for normoglycemia; ameliorated neuropathic signs. | Not specified | (Monfrini et al., 2017) |
| MSC2 (subset) | Cells | 0.5-1 ×10^6^ cells/mouse, i.p. | STZ-induced (50 mg/kg, i.p.), C57BL/6J (B6) mice | Not specified | MSC2 improved behavioral outcomes; conventional MSCs improved thermal but not mechanical stimuli. | Reduced serum proinflammatory cytokine levels. | (Waterman et al., 2012) |
| ADSCs | CM | CM administered every 2 wk for 4 doses | BKS *db/db* mice (BKS.Cg-*m*^+/+^*Lepr*^db^/J) | Not specified | Prevention of lower limb amputation risk; accelerated wound healing. | Not specified | (De Gregorio et al., 2020) |
| EPCs | Cells | 1×10^6^ cells | STZ-induced (150 mg/kg, i.p.), C57BL/6J mice (6 wk) | Not specified | Restored nerve function, perfusion, and capillary density, while boosting local angiogenic and neurotrophic factors. | Enhanced neovascularization via long term engraftment in nerves/vasa nervorum and paracrine effects. | (Jeong et al., 2009) |
| DPSCs | Cells | 1 × 10^6^ cells, i.m. | STZ-induced SD rats (6 wk) | Conditioned medium promoted DRG neurite outgrowth; increased SC viability and myelin related protein expression. | Improved NCV, pain perception, neurovascular density; increased myelin thickness and area. | Not specified | (Omi et al., 2017) |
| hDPSCs | Cells | 1 × 10^5^ cells/limb in 0.2 mL saline at 10 sites, unilateral hindlimb muscle | STZ-induced (150 mg/kg, i.p.), BALB/cAJcl-^nu/nu^ male nude mice (6 wk) | CM promoted DRG neurite outgrowth. | Improved delayed NCV, decreased blood flow, and increased sensory perception thresholds. | Not specified | (Hata et al., 2021) |
| hDPSCs | Cells | 1 × 10^5^ cells/limb, unilateral hindlimb skeletal muscle | STZ-induced (150 mg/kg, i.p.), BALB/cAJcl-^nu/nu^ male nude mice (6 wk) | Not specified | Efficacy in treating diabetic polyneuropathy via angiogenic and neurotrophic mechanisms of secreted factors. | Not specified | (Hata et al., 2020) |
| BMSCs | Cells/CM | 1×10^6^ BMSCs or MSC-CM | STZ-induced (80 mg/kg, i.p. for 3 d), C57BL/6J (B6) mice | Not specified | Reduced morphological/morphometric alterations in nerves of treated mice. | Increased anti-inflammatory cytokines IL-10 and TGF-β. | (Evangelista et al., 2018) |
| BMSCs | Cells | 5×10^7^ cells into hindlimb muscles | STZ-induced (75 mg/kg, i.p.), Wistar rats (8 wk) | Not specified | Restored NCV; increased vasa nervorum density; restored myelinated fiber ultrastructure. | Upregulated gene expression of angiogenesis-, neural function-, and myelination-related factors. | (Han et al., 2016) |
| PMSCs | Cells | 10^6^ PMSCs, i.m. at 5 sites/limb | Male *db/db* mice (BKS.Cg-*Dock7*^m+/+^*Lepr*^db^/Nju) | Improved nerve myelin lesions, promoted nerve regeneration, and activated SCs. | Improved DPN symptoms, nerve myelin lesions, nerve regeneration, and SC activation. | Regulation of Wnt signaling pathway. | (Pan et al., 2022) |
| BM-EPCs | Cells | AMD3100 (5 mg/kg, i.p.) + SDF-1α (1 μg) into left hind limb muscles twice | STZ-induced (60 mg/kg, i.p., daily for 5 d), Balb/C mice | Not specified | Restored sciatic vasa nervorum; improved impaired sciatic NCV. | Not specified | (Kim et al., 2013) |
| BMMNCs | Cells | 1 mL/site (1-5 cm in depth), i.m. at 50 sites (2 cm × 2 cm in intervals) in both thighs and legs | 168 patients with refractory DPN | Not specified | Significant post-transplant improvement in neuropathy signs/symptoms. | Not specified | (Mao et al., 2019) |
| ADSCs | Preconditioned Cells | Preconditioning with deferoxamine (150 or 400 μM, 48 h) | Not specified | Increased total antioxidant capacity of secretome; neuroprotective effects evaluated. | Not specified | HIF-1α activation leading to upregulation of VEGFα, angiopoietin-1, NGF, GDNF, neurotrophin-3, IL-4, IL-5. | (Oses et al., 2017) |
| MSC-like populations | Cells (PDA-002) | Low-dose (3×10^6^ cells) or high-dose (30×10^6^ cells), unilateral local injection | 26 patients with DPN | Not specified | Efficacy and safety evaluation for DPN treatment following unilateral local injection. | Not specified | (Gibbons et al., 2021) |
| BMSCs | Cells | 3×10^6^ cells in 1 mL, tail-vein | STZ-induced (220 mg/kg, i.p.), female Wistar rats | Not specified | Stimulated angiogenesis and sciatic nerve remyelination; improved behavioral outcomes. | Reduction of oxidative stress; promotion of nerve regeneration; modulation of inflammatory and neurotrophic factors; upregulation of S100 and VEGF expression. | (Mohamed et al., 2024) |
| BMSCs | Cells | 5 ×10^6^ or 1×10^7^ cells | STZ-induced, male SD rats | Not specified | Ameliorated oxidative stress, painful diabetic neuropathy, neurotrophic status, and angiogenesis. | Activation of GSK-3β/β-catenin signaling pathway in rats and SCs under high glucose. | (He et al., 2020) |

**Abbreviations:** ADSCs, adipose-derived stem cells; BDNF, brain-derived neurotrophic factor; BM-EPCs, bone marrow-derived endothelial progenitor cells; BMSCs, bone marrow-derived mesenchymal stem cells; BMMNCs, bone marrow mononuclear cells; CM, conditioned medium; DPN, diabetic peripheral neuropathy; DPSCs, dental pulp stem cells; DRG, dorsal root ganglion; EPCs, endothelial progenitor cells; GDNF, glial cell line-derived neurotrophic factor; GSK-3β, glycogen synthase kinase-3 beta; HFD, high-fat diet; HIF-1α, hypoxia-inducible factor 1-alpha; hDPSCs, human dental pulp stem cells; i.m., intramuscular; i.p., intraperitoneal; IENF, intraepidermal nerve fiber density; IL, interleukin; NCV, nerve conduction velocity; NF-κB, nuclear factor kappa-light-chain-enhancer of activated B cells; NGF, nerve growth factor; NT-3, neurotrophin-3; PMSCs, placenta-derived mesenchymal stem cells; SCs, Schwann cells; SD rats, Sprague-Dawley rats; STZ, streptozotocin; TGF-β, transforming growth factor-beta; VEGFα, vascular endothelial growth factor alpha; wk, weeks.
