# Supplementary material for: From pathophysiology to therapy: molecular mechanisms of stem cell and extracellular vesicle-mediated repair in diabetic peripheral neuropathy
Source: Front Cell Dev Biol. 2026 Jun 16;14:1854350. doi: 10.3389/fcell.2026.1854350 (PMC13314850; doi:10.3389/fcell.2026.1854350)
Supplement: Supplementary file 2 [file Table2.docx]

**Table S2. Therapeutic effects and mechanisms of EVs in DPN.**

| **Cell Origin** | **Product Type** | **Dose & Regimen** | **Disease Model** | ***In Vitro* Effects** | ***In Vivo* Outcomes** | **Proposed Mechanism** | **Ref.** |
| --- | --- | --- | --- | --- | --- | --- | --- |
| NSCs | EVs | 20 mg/kg, i.p. | STZ-induced (100 mg/kg, i.p.), male C57BL/6J mice (4–6 mo) | Microglia shift from M1 to M2 phenotype; reduced inflammatory cytokines; restored neuronal regulatory proteins. | Improved pain-related behavior; reduced histopathological nerve damage. | Immunomodulation and neural repair via WNT5a/TRPV1 axis regulation. | (Chen et al., 2025) |
| ADSCs | EVs | 10 µg/100 µL, s.c., weekly for 6 wk | STZ-induced (60 mg/kg, i.p.), SD rats (2 mo, 180-200 g) | Inhibited SC apoptosis;  promoted proliferation under high-glucose. | Promoted angiogenesis in DPN model. | miR-130a-3p from ADSC-EVs activated NRF2/HIF1α/ACTA1 axis via down-regulating DNMT1. | (Chen et al., 2023) |
| MSCs | Exos (engineered) | Exo-naïve or exo-146a, tail vein, weekly for 4 wk | Male *db/db* mice (BKS.Cg-*m*^+/+^*Lepr*^db^/J) | Not specified | Exo-146a induced faster improvement in NCV and thermal/mechanical thresholds vs. naïve Exos. | Suppression of peripheral blood inflammatory monocytes via TLR4/NF-κB pathway inhibition. | (Fan et al., 2021) |
| BMSC | Exos | 1×10^9^ particles, i.v., weekly for 8 wk | Male *db/db* mice (C57BLKS/J-leprdb/leprdb) | Not specified | Improved neurological recovery and NCV. | Elevated TOMM20 and Nrf2/HO-1; reduced MDA; increased GSH, SOD activity, and MMP. | (Shan et al., 2022) |
| SCs (engineered) | EVs (214-EVs) | 2 × 10¹⁰ particles/mouse, tail vein, once weekly for 8 wk | HFD + STZ-induced (75 & 50 mg/kg), male C57BL/6 mice (10 wk) | Not specified | Improved motor/sensory NCV and thermal latency; enhanced IENF density, axonal diameter, and myelin thickness. | Suppressed neuroinflammation by reducing CD68⁺ macrophages and inactivating TLR4/NF-κB signaling. | (Wang et al., 2025a) |
| MSCs | Exos | Tail vein, weekly for 8 wk | *db/db* mice (BKS. Cg-*m*^+/+^*Lepr*^db^/J) | Decreased M1 macrophage phenotype; increased M2 markers. | Decreased thermal and mechanical sensitivity thresholds; increased NCV, blood vessels density, intraepidermal nerve fiber density, myelin thickness and axon diameter. | miRNA mediated targeting of TLR4/NF-κB signaling pathway. | (Fan et al., 2020) |
| SCs (under HG) | Exos | 1 × 10⁸ particles (HG-Exos), daily injection for 7 d | Male *db/db* mice (BKS.Cg-*m*^+/+^*Lepr*^db^/J) | Inhibited axonal growth. | Impaired NCV; induced mechanical/thermal hypoesthesia. | Enriched miR-28, -31a, and -130a. | (Jia et al., 2018) |
| huMSCs | Exos | Not specified | Not specified | Promoted keratinocyte proliferation/migration; inhibited hyperglycemia-induced ferroptosis via miR-548ai/miR-660. | Enhanced cutaneous wound healing in diabetic mice. | Repression of ACSL4-mediated ferroptosis. | (Yang et al., 2024) |
| Pericytes | EVs (PC-NVs) | 0.5, 1, or 5 µg in 20 µl HBS, applied to penis on days -3 and 0 | STZ-induced (50 mg/kg, i.p.), male C57BL/6J mice (8 wk) | Induced endothelial proliferation and migration; reduced apoptosis under diabetic conditions. | Induced neural regeneration in DRG and MPG explants. | Activated MAPK and PI3K/Akt pathways; suppressed p53 signaling in an Lcn2-dependent manner. | (Anita et al., 2022) |
| SCs | Exos | 4 × 10¹⁰ particles, tail vein, biweekly for 8 wk | Male *db/db* mice (BKS.Cg-*m*^+/+^*Lepr*^db^/J) | Promoted neurite outgrowth and SC migration under high glucose. | Improved sciatic NCV; increased thermal and mechanical sensitivity. | Reversed diabetes-induced reduction of miR-21, -27a, -146a; modulated SEMA6A, RhoA, PTEN, and NF-κB expression. | (Wang et al., 2020) |
| SCs/Serum | Exos | Not specified | STZ-induced (50 mg/kg, i.p.), SD rats (7 wk) | Downregulated miR-21 under high glucose; promoted SC apoptosis and neurite outgrowth; inhibited proliferation. | Decreased miR-21 levels in serum exosomes from DPN rats. | Participated in neurite outgrowth via AKT signaling pathway. | (Liu et al., 2022) |
| MSCs | EVs (engineered) | MEVs (1 × 10^6^, 1 × 10^7^, or 1 × 10^8^ particles) i.v. every 4 wk | *db/db* mice | Aalleviated photoreceptor injury. | Enhanced retinal repair, attenuated dysfunction and photoreceptor loss. | USP25-mediated inhibition of hyperglycemia-induced CRYAA ubiquitination. | (Sun et al., 2025) |
| BMSCs | Exos | Exos (1 × 10^6^) mixed with liposomes (5 × 10^5^) | STZ-induced (65 mg/kg, i.p.), female Wistar rats | Exos internalization in neural cell lines; protection against oxidative stress. | Normalized neurological/muscular functions; controlled hyperglycemia/weight loss; attenuated tissue damage. | Not specified | (Singh et al., 2021) |
| SCs | Exos | SCs were treated with PF at low, middle, and high doses (referred to as PF1, PF10, PF100 groups) | Not specified | PF-treated SC-EXOs alleviated ER stress, improved ER morphology, and reduced apoptosis in HG-treated DRGn. | Not specified | PF-treated SC-EXOs significantly inhibited the IRE1α pathway (reduced GRP78, IRE1α, p-IRE1α). | (Zhu et al., 2021) |

**Abbreviations:** ACSL4, acyl-CoA synthetase long-chain family member 4; ACTA1, actin alpha 1, skeletal muscle; ADSCs, adipose-derived stem cells; AKT, protein kinase B; BMSCs, bone marrow-derived mesenchymal stem cells; CRYAA, crystallin alpha A; DNMT1, DNA methyltransferase 1; DPN, diabetic peripheral neuropathy; DRG, dorsal root ganglion; ER, endoplasmic reticulum; EVs, extracellular vesicles; Exos, exosomes; GRP78, Glucose-regulated protein 78; GSH, glutathione; HG, high glucose; HFD, high-fat diet; HIF1α, hypoxia-inducible factor 1-alpha; huMSCs, human umbilical cord mesenchymal stem cells; IENF, intraepidermal nerve fiber density; i.p., intraperitoneal; IRE1α, Inositol-requiring enzyme 1 alpha; i.v., intravenous; MAPK, mitogen-activated protein kinase; MDA, malondialdehyde; MMP, mitochondrial membrane potential; MPG, major pelvic ganglion; MSCs, mesenchymal stem cells; NCV, nerve conduction velocity; NF-κB, nuclear factor kappa-light-chain-enhancer of activated B cells; Nrf2, nuclear factor erythroid 2–related factor 2; NSCs, neural stem cells; PC-NVs, pericyte-derived nanovesicles; PF, Paeoniflorin; PI3K, phosphoinositide 3-kinase; p-IRE1α, Phosphorylated inositol-requiring enzyme 1 alpha; PTEN, phosphatase and tensin homolog; RhoA, Ras homolog family member A; s.c., subcutaneous; SCs, Schwann cells; SD rats, Sprague-Dawley rats; SEMA6A, semaphorin 6A; SOD, superoxide dismutase; STZ, streptozotocin; TLR4, Toll-like receptor 4; TOMM20, translocase of outer mitochondrial membrane 20; TRPV1, transient receptor potential vanilloid 1; USP25, ubiquitin-specific peptidase 25; WNT5a, Wnt family member 5A; wk, weeks.
